# Outcomes of the Expectant Management of 10 Cesarean Scar Pregnancy Cases in Patients Who Refused the Termination of Pregnancy

**DOI:** 10.7759/cureus.48921

**Published:** 2023-11-16

**Authors:** Atif Fazari, Parveen Bhanu Mohammed, Asma Fahad

**Affiliations:** 1 Faculty of Medicine, University of Medical Sciences and Technology, Khartoum, SDN; 2 Obstetrics and Gynecology, Latifa Hospital, Dubai Academic Health Corporation, Dubai, ARE; 3 Obstetrics, Latifa Hospital, Dubai Academic Health Corporation, Dubai, ARE

**Keywords:** obstetric outcome, cesarean hysterectomy, placenta accreta spectrum, doppler sonography, prenatal ultrasound mapping, multidisciplinary team, clinical expectant management, caesarean scar pregnancy

## Abstract

Objective: Expectant management of cesarean scar pregnancy (CSP) in patients who refuse termination of pregnancy and continue with placenta accreta spectrum (PAS) is possible with multidisciplinary care and careful monitoring in a tertiary care center. Doctors with the relevant expertise in managing PAS use highly accurate ultrasound as a tool to diagnose, monitor, and manage this disorder, which enables them to determine appropriate surgical strategies and techniques to achieve optimum maternal and fetal outcomes with minimal blood loss and no major maternal mortality and morbidity. In this study, we aim to evaluate expectant management in such patients.

Materials and methods: This is a retrospective study of 10 patients with a previous history of a uterine scar. Diagnosed with CSP in the first trimester, they refused to terminate their pregnancy and continued with PAS. We studied them over a period of four years from 2018 to 2022 and managed them at Latifa Hospital, Dubai, UAE.

Results: Of the 10 patients, nine delivered in the third trimester (around 34 weeks gestation), seven underwent elective surgery, and three underwent emergency surgery. Four patients were exogenous cases and six were endogenous cases at diagnosis during early gestation. Seven patients had a cesarean hysterectomy, and three (with focal placenta accreta) had uterine wall reconstruction surgery. Four patients needed blood transfusions. The average duration of surgery was between 2.5 and 5 hours. There were no miscarriages, no maternal and neonatal deaths, and no significant obstetric complications such as rupture of the uterus or major obstetric hemorrhage.

Conclusion: Even though CSP is a potentially life-threatening condition because of serious complications such as PAS if continued, expectant management is possible under multidisciplinary care where the team strictly adheres to clinical protocols and accurate surgery to reduce obstetric hemorrhage.

## Introduction

Cesarean scar pregnancy (CSP) refers to a pregnancy that implants in or on a defect or myometrial gap in a previous uterine scar. Managing CSP can be challenging, and if left unrecognized or untreated, or if the patient refuses to terminate the pregnancy, it can progress to a viable pregnancy. Furthermore, it can transform into the morbidly adherent placenta (MAP) or form part of the placenta accreta spectrum (PAS), both of which carry significant risks, including major obstetric hemorrhage, peripartum hysterectomy, and maternal morbidity and mortality. These two latter conditions share similar pathology and histology [1,2].

There are primarily two types of CSP. In type 1 CSP (endogenous), the pregnancy is entirely located within and continues to grow inside the uterine cavity, deep in the myometrium, but without protruding beyond the serosa. Type 1 CSP has a higher potential to reach a viable gestation, possibly up to the third trimester, and is associated with complications such as placenta accreta or placenta increta [1,3]. In type 2 CSP (exogenous), the pregnancy is located mainly in the lower uterine segment and extends outside the serosa, growing toward the bladder. It can lead to complications such as uterine rupture and intraperitoneal bleeding, posing a significant risk of mortality and morbidity [1,3].

There is also the rare type 3 CSP (mixed) that exhibits characteristics of both types 1 and 2 CSP.

Transvaginal ultrasound is highly accurate for the prenatal diagnosis, monitoring, and management of CSP evolving into PAS. Ultrasound findings can provide valuable information about the depth of villous invasion and vascularity and can guide surgical strategies while predicting clinical outcomes [4].

Characteristic ultrasound findings of PAS include the absence of the retroplacental hypoechoic clear space, reduced uterine wall thickness, with myometrial thickness measuring less than 3 mm, and increased vascularity surrounding the myometrium. This can manifest as numerous large blood vessels and may lead to an irregular bladder wall with extensive associated vascularity [5].

Other findings include Fazari’s triad or Fazari’s sign (stair-stepped appearance). Several features characterize this sign, including the ballooning of the lower segment because of the presence of the entire placenta; a complete absence of the uterine wall, which is replaced by a vascular bed; and the placenta badly adhering to the urinary bladder, with penetration into the bladder space and its vasculature [5,6].

“Equal sign”-crossing or bridging vessels is another sign seen in PAS. This phenomenon is defined as the presence of two parallel vessels, indicating neovascularization, as observed through color Doppler imaging. These vessels typically appear over the ureterovesical junction and extend toward the bladder wall, forming interconnecting bridging vessels that run perpendicular to both ureterovesical walls [4].

Bulging of the urinary bladder wall is also a characteristic ultrasound finding of PAS; it refers to the noticeable protrusion or swelling of the urinary bladder wall, often associated with the presence and infiltration of the placenta [5,6].

## Materials and methods

This is a retrospective study of 10 patients with a previous history of uterine scar, with CSP diagnosed in the first trimester, who refused to terminate their pregnancy and continued with MAP or PAS. We included cases over a four-year period from 2018 to 2022.

We collected data using our Epic electronic data system.

After diagnosing CSP, we followed the patients in the maternal-fetal medicine unit with serial ultrasound scans (2D USS) and Doppler sonography. We mapped the placenta with the retroplacental myometrium and bladder when the bladder was partially full. We evaluated the progression of the PAS to look for and observe the aforementioned characteristic USS features of PAS. We counseled patients thoroughly regarding potential complications of major obstetric hemorrhage, blood transfusions, and hysterectomy. We obtained their high-risk informed consent and planned their surgery, that is, a classical cesarean section with a cesarean hysterectomy at 34-35 weeks.

We performed surgery at Latifa Hospital in Dubai, United Arab Emirates, and our multidisciplinary team of two senior OB-GYN consultants, a senior anesthesiologist, a urologist (who performed preoperative ureteral stenting to avoid urinary tract injuries), and a vascular surgeon on standby strictly followed protocols during surgery. We also had equipped blood bank services and an intensive care unit (ICU). We performed a midline longitudinal incision for a classical cesarean section and a cesarean hysterectomy if the placenta was not separated. We also performed major artery ligation in some cases. We used accurate surgical strategies and techniques involving meticulous adhesiolysis after the introduction of a neat dissection technique - Birds’ picking seeds’ technique [6] - as per prenatal USS vascular mapping to reduce obstetric hemorrhage.

We analyzed the results using Statistical Package for the Social Sciences (IBM SPSS Statistics for Windows, IBM Corp., Version 26.0, Armonk, NY), depending on factors such as gestational age at diagnosis, CSP type, gravida, parity, previous cesarean section history, gestational weeks at termination, amount of blood loss, duration of surgery, and correlating the USS findings with surgical findings to assess obstetric and neonatal outcomes and complications in expert hands.

## Results

Of the 10 patients, nine (90%, 9/10) delivered in the third trimester (up to an average of 34 weeks gestation), and one (10%, 1/10) delivered at 27 weeks gestation. Of the patients in the third trimester, two (20%, 2/10) delivered at 30 and 31 weeks gestation, respectively. Seven (70%, 7/10) underwent elective surgery, and three (30%, 3/10) underwent emergency surgery. Four (40%, 4/10) were exogenous (type 2 CSP), and six (60%, 6/10) were endogenous (type 1 CSP) at diagnosis during early gestation. Seven (70%, 7/10) of the 10 patients had a cesarean hysterectomy, and three (30%, 3/10) (with focal placenta accreta) had uterine wall reconstruction surgery. Three patients (30%, 3/10) had major artery ligation. Four patients (40%, 4/10) needed a blood transfusion (on average a maximum of two to four units); six (60%, 6/10) did not. The average duration of surgery was between 2.5 and 5 hours, with an overall average of three hours. We admitted four patients (40%, 4/10) to ICU. We diagnosed all 10 patients pathologically and histologically as having placenta accreta, of which three (30%, 3/10) had focal accreta and did not undergo hysterectomy. In all cases, there was an accurate correlation between characteristic USS features and surgical findings. There were no miscarriages, no maternal deaths, and no significant obstetric complications such as rupture of the uterus or major obstetric hemorrhage. We found no statistical difference in patients regarding gravidity, parity, and previous cesarean section. Considering neonatal outcomes, we admitted three neonates to the neonatal intensive care unit (NICU) (Table 1).

**Table 1:** Summary of Results 1 = Loss of hypoechoic line unmeasurable space; 2 = Vascularity; 3 = Equal signs - crossing or bridging vessels; 4 = Bulging of urinary bladder wall; 5 = Fazari’s sign; Exo = Exogenous; Endo = Endogenous; HYS = hysterectomy; UWRS = uterine wall reconstruction surgery; UB = urinary bladder; Em = emergency; El = elective; LSCS = Lower-segment cesarean section; CSP = Cesarean scar pregnancy; GA = Gestational age

Serial Number	No. of Previous LSCS	Type of CSP	GA at Diagnosis	Follow-Up Scan	GA at Surgery	Intervention	Surgery	Blood Transfusion	Surgery Duration	Finding
1	4	Exo	6	1,2,3,4,5	30	Em	HYS	2	3 hrs	Crossing vessels to UB
2	2	Exo	8	1,2,3,4,5	33	El	HYS	0	4 hrs	Crossing vessels to UB
3	4	Exo	7	1,2,3,4,5	34	El	HYS	0	2.5 hrs	Crossing vessels to UB
4	1	Exo	6	1,2,3,4,5	33	El	HYS	0	4.5 hrs	Crossing vessels to UB
5	3	Endo	9	1,2,3,4,5	27	Em	HYS	4	3 hrs	Crossing vessels to UB
6	1	Endo	8	1,2,4	35	El	UWRS	2	5 hrs	Free UB wall
7	2	Endo	6	1,2,3,4,5	31	Em	UWRS	0	3 hrs	Crossing vessels to UB
8	3	Endo	10	1,2,3,4,5	34	El	HYS	0	3 hrs	Crossing vessels to UB
9	2	Endo	8	1,2,4	34	El	UWRS	2	2.5 hrs	Free UB wall
10	4	Endo	7	1,2,3,4,5	34	El	HYS	0	3.5 hrs	Crossing vessels to UB

## Discussion

CSP is often considered a precursor to PAS, and the recent rise in its incidence may be attributed to the global increase in cesarean section rates, particularly in the Middle East because of cultural factors [3,7-9]. CSP and PAS are associated with significant maternal and fetal morbidity and mortality, including complications such as major obstetric hemorrhage, hysterectomy, surgical issues, preterm birth, and fetal loss [1-3,5,8,10-12]. The escalating occurrence of these conditions worldwide, especially in the Middle East, underscores the need for clear protocols and strategies for their diagnosis, monitoring, management, and follow-up in specialized tertiary care centers [5,6].

In cases where doctors offer expectant management, we recommend a cesarean delivery between 34 and 35 weeks gestation for women with CSP. When MAP complicates CSP, the most common therapeutic approach is uterine removal, with the procedure ideally performed in a tertiary care unit by an experienced surgeon supported by senior anesthetists, urologists, and vascular surgeons, with immediate access to an ICU and blood bank services for blood transfusion and blood products [5,6].

There is a lack of consensus on the best practices for expectant management of CSP, and considerable global variation exists. Some researchers have reported significant maternal morbidity, primarily related to hemorrhage and cesarean hysterectomy because of PAS in cases managed expectantly, whereas others have reported high live birth rates [13,14]. Limited data are available regarding the management and maternal outcomes of expectant management of CSP progressing to PAS or MAP, with most of the literature consisting of case reports and retrospective cohort studies [1,12].

In our research, we focused on a small population of women in the UAE who refused termination of pregnancy because of cultural factors and chose expectant management, despite being aware of the risks associated with CSP and PAS. The sonographic signs and outcomes we examined are valuable for counseling women undergoing expectant management about their prognosis. We conducted an online search of the PubMed and Cochrane Library databases to gather studies on women diagnosed with CSP who were managed expectantly and continued to develop PAS, allowing us to compare our findings with existing research [1,3].

Our research aligns with several previous studies that support expectant management of CSP progressing to MAP or PAS when managed in specialized tertiary care centers. These studies suggest lower mortality and morbidity rates, highlighting the connection between CSP and PAS or MAP, especially CSP type 1 (endogenous) and type 2 (exogenous), although they are limited by their small study populations [7-9,12].

On the contrary, in a recent study, the researchers presented a different perspective. They found that patients who underwent expectant management of CSP had a 20.1% miscarriage rate, an 8.3% fetal death rate, and a 9.9% uterine rupture rate in the first or second trimester. Among patients who progressed to the third trimester, 70% developed placenta percreta, and 40% experienced severe bleeding. Additionally, 25.8% had term deliveries, 41.8% experienced preterm births, and 13.9% delivered before 34 weeks gestation. Doctors performed hysterectomies in 52.6% of cases [14].

In our study, there is a lack of evidence regarding the diagnosis and expectant management of CSP, continuing as PAS or MAP in low-resource settings. We offered termination of pregnancy to the majority of our patients diagnosed with CSP in the first trimester in view of the association of significant maternal morbidity with expectant management. However, the potential to obtain a live birth, with expectant management in some CSP cases resulting in good maternal outcomes, warrants consideration of offering expectant management as an alternative treatment. One systematic review of the research showed that 57% of women had live births, whereas 63% had a hysterectomy for PAS or MAP and for uterine rupture [12,14].

Although it's a small sample size, our study demonstrated positive outcomes because we managed our patients in a tertiary hospital setup, considered a placenta accreta center of excellence with highly equipped facilities, including a blood bank and ICU, and a highly trained and experienced management team. Ninety percent (9/10) of our patients delivered in the third trimester (around 34 weeks gestation), with 10% (1/10) delivering in the second trimester at 27 weeks (preterm birth). Seventy percent (7/10) required a cesarean hysterectomy, whereas 30% (3/10) retained the uterus and underwent uterine wall reconstruction surgery for focal accreta. Among all surgeries, 30% (3/10) were emergencies, and 70% (7/10) were elective. In our study, there was a 100% (10/10) live birth rate, although 30% (3/10) of neonates required admission to the NICU because of prematurity. Forty percent (4/10) of our patients needed blood transfusions. We had a distribution of 40% (4/10) exogenous and 60% (6/10) endogenous types of CSP progressing to MAP or PAS. Importantly, we did not report any miscarriages, maternal deaths, or significant obstetric complications such as uterine rupture or major obstetric hemorrhage.

Effective management of CSP and PAS, whether expectant or not, necessitates the establishment of clear strategies for early diagnosis, referral, patient selection, follow-up, care, and management. Surgical options should be considered based on available expertise and individual and geographical circumstances. Preoperative assessment, counseling, intraoperative planning, postoperative follow-up, and education are crucial for successful outcomes [5].

However, larger-scale studies and more research are needed to draw definitive conclusions and assess the risks associated with expectant management of CSP and PAS or MAP [6,15].

There were several limitations in our study. First, this study was a retrospective study. Second, it is a single-center study and the case number was limited. Also, our study included patients who opted to continue with CSP due to social, cultural, and religious beliefs. More studies need to be done on this topic.

## Conclusions

Even though CSP is a potentially life-threatening condition because of serious complications such as PAS if continued (especially in CSP type 2), expectant management is possible with close monitoring in a tertiary care center under an expert multidisciplinary team that strictly adheres to clinical protocols during the antenatal period and performs accurate surgery to reduce obstetric hemorrhage and overall mortality and morbidity. Additional research is required to establish conclusive findings and assess the potential risks associated with expectant management.
